# Efficient simultaneous double DNA knock-in in murine embryonic stem cells by CRISPR/Cas9 ribonucleoprotein-mediated circular plasmid targeting for generating gene-manipulated mice

**DOI:** 10.1038/s41598-022-26107-z

**Published:** 2022-12-13

**Authors:** Manabu Ozawa, Jumpei Taguchi, Kento Katsuma, Yu Ishikawa-Yamauchi, Mio Kikuchi, Reiko Sakamoto, Yasuhiro Yamada, Masahito Ikawa

**Affiliations:** 1 Laboratory of Reproductive Systems Biology, Center for Experimental Medicine and Systems Biology, The Institute of Medical Science, The University of Tokyo, Tokyo, 108-8639 Japan; 2 Division of Stem Cell Pathology, Center for Experimental Medicine and Systems Biology, The Institute of Medical Science, The University of Tokyo, Tokyo, 108-8639 Japan; 3 Research Institute for Microbial Diseases, Osaka University, Osaka, 565-0871 Japan

**Keywords:** CRISPR-Cas9 genome editing, Embryonic stem cells, Genetic transduction, Genetic engineering

## Abstract

Gene targeting of embryonic stem (ES) cells followed by chimera production has been conventionally used for developing gene-manipulated mice. Although direct knock-in (KI) using murine zygote via CRISPR/Cas9-mediated genome editing has been reported, ES cell targeting still has merits, e.g., high throughput work can be performed in vitro. In this study, we first compared the KI efficiency of mouse ES cells with CRISPR/Cas9 expression vector and ribonucleoprotein (RNP), and confirmed that KI efficiency was significantly increased by using RNP. Using CRISPR/Cas9 RNP and circular plasmid with homologous arms as a targeting vector, knock-in within ES cell clones could be obtained efficiently without drug selection, thus potentially shortening the vector construction or cell culture period. Moreover, by incorporating a drug-resistant cassette into the targeting vectors, double DNA KI can be simultaneously achieved at high efficiency by a single electroporation. This technique will help to facilitate the production of genetically modified mouse models that are fundamental for exploring topics related to human and mammalian biology.

## Introduction

The development of gene-manipulated mouse models and the determination of their phenotypic features is a robust technology that is used to precisely analyze gene functions or physiological behaviors of specific cells in the animal body. Several important biological phenomena have been uncovered by using various such gene-manipulated animal models. Locus-specific exogenous gene integration technique, termed gene knock-in (KI), has several merits, e.g., an accurate integration site, controlled copy number, and regulated KI gene expression, which more closely resembles the locus-specific gene expression patterns of particular cell types^1,2^. The conventional way to develop gene KI mouse models is to use the embryonic stem cell (ES cells) targeting technique followed by the ES cell injection to the preimplantation embryo to produce chimeras^3–5^.

The induction of double-strand breaks in the genome increases the integration efficiency of the transgene by homologous recombination in mammalian cells^6^. More recently, the introduction of site-specific endonucleases such as ZFN^7,8^, TALEN^9,10^, or CRISPR/Cas9^11^, which induce double-strand breaks at specified sequences, have made it possible to modify the zygote genome directly. Among these genome editing techniques, CRISPR/Cas9 is now used in many laboratories as a powerful tool for embryo genome editing due to its simplicity and accuracy. CRISPR/Cas9-mediated locus-specific gene manipulation using mouse embryos was first reported by introducing single-guide RNA and Cas9 mRNA into the pronucleus of fertilized zygotes using micromanipulation^11^, and several papers have reported that analysis of F0 mice generated by CRISPR/Cas9-mediated zygote genome editing revealed several important gene functions in some fields such as sleeping physiology^12,13^ or spermatogenesis^14^. Not only for induction of indel mutation^11^, but site-specific short nucleotide KI such as single nucleotide replacements^11^ or peptide tags insertion^15^ into murine zygotes can also accomplish by using single-strand oligonucleotide (ssODN) as KI donor via homology-directed repair. In addition to the short fragment KI, DNA sequences longer than several kilo-base pairs (kbp) can be integrated into the zygote genome by CRISPR/Cas9-mediated genome editing using double-strand plasmid^15,16^, long single-strand DNA (lssDNA)^17,18^, or adeno-associated virus (AAV) as KI donors^19–21^.

Although zygote genome editing using several mouse strains such as C3H/HeJ^22^ or B6D2F1^11,15^ has been reported, the C57BL/6 strain is commonly used because of its flexibility in vitro handling, including hyper ovulation, in vitro fertilization, or in vitro embryo culture, as well as pure genetic background. On the other hand, zygote genome editing is still challenging in particular strains, such as BALB/c, which is an important strain for specific research areas such as immunology, because of their lower response to hyper ovulation or more susceptibility to in vitro embryo manipulations^23^.

Compared to the direct zygote genome edition, ES cell targeting is believed to have still several advantages for making gene-manipulated mouse models, e.g., high throughput genotyping screening can be performed in vitro*,* or ES cell lines established from a variety of inbred strains, including BALB/c^24^, DBA2^25^ or C57BL/6, can be used for gene manipulation. Another considerable merit is that establishing KI ES cell clones having multiple exogenous genes, e.g., multi-fluorescent reporters, or CreERT2 and loxP-STOP-loxP, at different genomic loci is possible by performing sequential KI^26^. However, repetitive gene targeting takes time, and the pluripotency of ES cells is known to decrease with longer culture periods^27^. Therefore, there is a need to develop a highly efficient technology that can KI long DNA fragments at multiple loci in a single gene transfer.

In this study, we describe a simple and highly efficient CRISPR/Cas9-mediated KI method in mouse ES cells for developing gene-manipulated mouse models. A single gene KI can be achieved efficiently without drug selection by using a circular plasmid as a targeting vector accompanied by KI site-specific induction of double-strand break via CRISPR/Cas9. We confirmed that this KI method was applicable in various chromosomal loci in various ES cell lines including inbred C57BL/6, BALB/c, or hybrid B6-129 F1. Furthermore, by incorporating the drug-resistant cassette in the targeting vectors, double KI at different genomic loci can be achieved at high efficiency, with > 60%, by a single electroporation.

## Results

### Integration of linearized DNA fragments occurs mainly randomly into the ES cell genome

The *Rosa26* locus is known as a safe harbor locus for stable gene expression^28^ and is frequently used for the development of KI mice with a fluorescein reporter^29,30^ or Cre/loxP conditional recombination^26,31^. Thus, we used this locus for determining homologous recombination-based KI using large-sized donor DNA. As linearized plasmids are routinely utilized as DNA donors for conventional gene targeting, we first compared the frequency of DNA integration into the genome between linearized or circular vectors. Linearized or circular plasmid carrying a 1850 bp CAG-EGFP cassette flanked by both the homology arms (5′ and 3′ of 962 and 1006 bp, respectively) of the *Rosa26* locus (pR26-CE) were transduced into ES cells via electroporation (Supplementary Fig. S1A,B). Seven to 10 days later, the expression of EGFP was determined via microscopy or flow cytometry. Although the number of EGFP-positive cells was quite low for both transductions (0.7 ± 0.1% and 0.4 ± 0.1% in the linearized and circular vectors, respectively), the percentage of EGFP-positive cells significantly increased when the linearized vector was used (Supplementary Fig. S1C,D).

Next, we selected ES cells in which the linearized vector was integrated into the genome via drug resistance and verified whether the genomic integration of the vector was homology arm-dependent site-specific KI. We transduced ES cells with linearized pR26-CE-PN, which contained PGK-NeoR cassette subcloned in the 3′ end of CAG-EGFP in pR26-CE, (Supplementary Fig. S2A), added G418 to the culture medium 24 h post-electroporation, and cultured the cells for an additional 7–10 days to select stable drug-resistant clones. Following G418 selection, almost all ES cell colonies were EGFP-positive (Supplementary Fig. S2B). Analysis of each colony for *Rosa26* locus-specific KI via genomic PCR showed that none of the 47 independent clones contained any KI specific bands either on the 5′ or 3′ side (Supplementary Fig. S2C). These results indicated that integration of linearized targeting vector into the genome is mainly random and is not site-specific although the targeting vector carried homologous arms, and circular plasmids, which are thought to have a lower frequency of random genomic integration than a linearized plasmid, were used as targeting vectors hereafter.

### CRISPR/Cas9 ribonucleoprotein (RNP)-mediated circular plasmid integration into the ES cell genome was highly locus-specific

**Figure 1:**
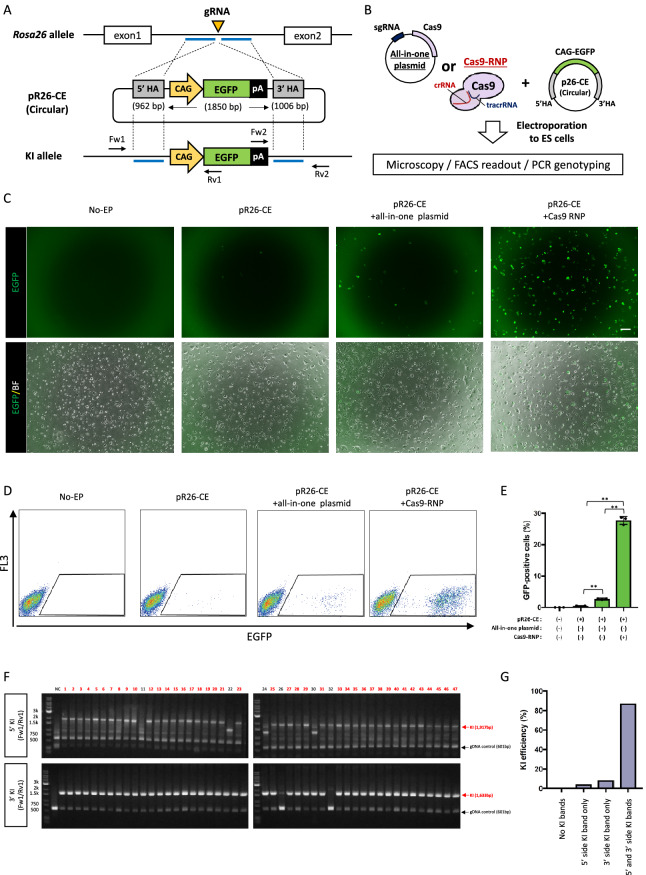
CRISPR/Cas9 ribonucleoprotein (RNP)-mediated circular plasmid integration into ES cell genome is highly locus-specific. (**A**,**B**) Schematic representations of circular plasmid introduction strategies. Linearized vector containing CAG-EGFP cassette flanked by approximately 1 kbp next to the gRNA target was transduced into ES cells via electroporation (**A**). Circular plasmid as a targeting vector was introduced into ES cells with CRISPR/Cas9 expression vector (left) or CRISPR/Cas9 ribonucleoprotein (RNP, right) by single electroporation (**B**). (**C**) Representative ES cell colonies under a fluorescent microscope. Top panels show EGFP images and bottom panels show merged images of EGFP and bright field. Left: no electroporation (No-EP); middle-left: transduction of targeting vector-only; middle-right: transduction of targeting vector with CRISPR/Cas9 all-in-one plasmid; right: transduction of targeting vector with CRISPR/Cas9 RNP. Scale bar, 50 µm. (**D**) Flow cytometry of ES cells. Gate represents EGFP-positive fraction. (**E**) EGFP-positive cell ratios are shown as mean ± SEM. The asterisk depicts a significant difference (n = 3, P < 0.01). (**F**,**G**) Genomic PCR analyses of EGFP-positive ES cell clones collected via flow cytometry. Gel images or KI ratios are shown in F or G, respectively. Red arrows in the upper or bottom panels indicate the 5′ or 3′ *Rosa26* KIs, respectively, and black arrows indicate genomic DNA (gDNA) PCR control. Both 5′ and 3′ PCR positive clones are indicated with red numbers. *NC* negative control using genomic DNA from wild-type B6 mouse tail.

Recent studies have indicated that Cas9-RNP-mediated genome editing exhibits lower cytotoxicity and higher efficiency of genomic rearrangement induction than that mediated by CRISPR/Cas9 expressing plasmids^32–34^. Thus, we next compared the efficiency of gene KI in ES cells with the all-in-one plasmid, which expressed both gRNA and Cas9 mRNA, or Cas9-RNP, which consisted of crRNA/tracrRNA/Cas9 protein. We transduced ES cells with the circular pR26-CE via electroporation with the CRISPR/Cas9 all-in-one plasmid or Cas9-RNP (Fig. 1A,B) and determined EGFP expression 7–10 days later. In this experiment, we did not use any drug-resistant cassettes for either positive or negative selection. Few EGFP-positive ES cells appeared when pR26-CE alone was transduced into ES cells (Fig. 1C,D). The introduction of pR26-CE and the all-in-one vector significantly increased the frequency of EGFP-positive ES cells, although the ratio remained low (2.7 ± 0.1%, Fig. 1D,E). By contrast, introducing pR26-CE and Cas9-RNP into ES cells increased the EGFP-positive cell ratio approximately tenfold compared with that found with the all-in-one plasmid (27.7 ± 0.7%, Fig. 1D,E). Next, ES cells that became EGFP-positive were sorted by FACS and passed into a fresh medium; cloned colonies were selected and used for KI genotyping by PCR. Of the selected clones, 41 out of 47 were positive for bands from both 5′ and 3′ KI (Fig. 1F), and the KI ratio was 87.2% (Fig. 1G). These results indicated that Cas9-RNP-mediated double-strand DNA KI in the *Rosa26* locus of ES cells became more evident than that found with the all-in-one plasmid. We then PCR amplified the *Rosa26* locus to see whether this plasmid KI was a homo- or hetero-integration event and analyzed 11 clones. One of these clones lacked the PCR amplicon (Supplementary Fig. S3A,B), whereas the remaining 10 clones had indel mutations (Supplementary Fig. S3C). This suggested that most cases in the *Rosa26* KI were hetero-integration KI events, whereas genetic rearrangement frequently occurs in both chromosomes after the Cas9-RNP induction.

### Cas9 RNP-mediated plasmid DNA KI is applicable to various loci in ES cells

**Figure 2:**
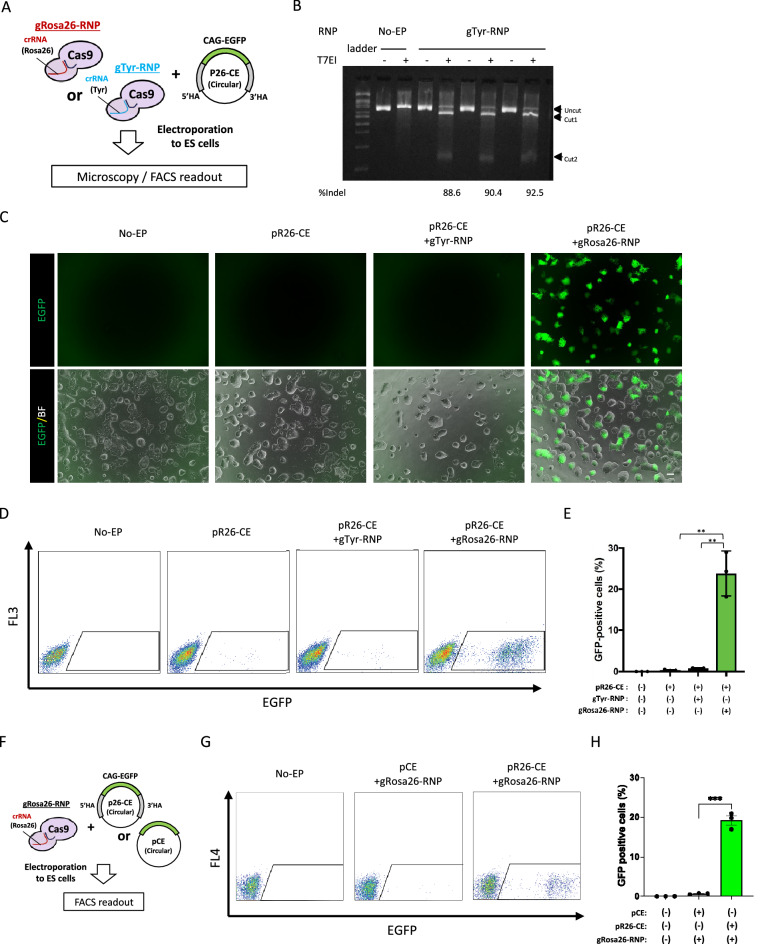
Cas9 RNP-mediated plasmid DNA KI is attributed to homologous recombination. (**A**) Schematic representations of Rosa26 locus or Tyrosinase locus genome editing by site-specific Cas9-RNP. Circular vector containing CAG-EGFP cassette flanked by 5’ and 3’ homology arms of Rosa26 was transduced with either gRosa26-RNP or gTyr-RNP into ES cells via electropolation. (**B**) T7 endonuclease I mismatch cleavage (T7EI) analysis for validating the efficiency of double-strand break induction by the tyrosinase locus-targeting CRISPR/Cas9 RNP. Upper, middle, or lower arrowheads indicate uncut wild-type genome, longer half of mutated genome, or short half of mutated genome, respectively. The numerical values on the bottom of the image represent induction ratios of indel mutation in the tyrosinase locus of targeted ES cells. Indel mutation ratios were calculated using the formula mentioned elsewhere (http://crispr.technology/resources/quantification.html). (**C**) Representative ES cell colonies under a fluorescent microscope. Top panels show EGFP images and bottom panels show merged images of EGFP and bright field. Left: no electroporation (No-EP); middle-left: transduction of pR26-CE plasmid-only; middle-right: transduction of pR26-CE targeting vector with tyrosinase locus-specific CRISPR/Cas9 RNP; right: transduction of pR26-CE targeting vector with *Rosa26* locus-specific CRISPR/Cas9 RNP. Scale bar, 50 µm. (**D**) Flow cytometry of ES cells. Gate represents EGFP-positive fraction. (**E**) GFP positive cell ratios are shown as mean ± SEM. The asterisk depicts a significant difference (n = 3, P < 0.01). (**F**) Schematic representations of Rosa26 locus genome editing by site-specific Cas9-RNP. The targeting vector with or without 5’ and 3’ homology arms of Rosa26 was transduced with gRosa26-RNP into ES cells via electroporation. (**G**) Flow cytometry of ES cells. Gate represents EGFP-positive fraction. (**H**) GFP positive cell ratios are shown as mean ± SEM. The asterisk depicts a significant difference (n = 3, P < 0.001).

Next, we determined whether the Cas9-RNP-mediated circular plasmid KI is attributed to homology arm-mediated homologous recombination or Cas9-RNP-induced double-strand break-site-specific integration without homologous recombination. First, Cas9-RNP that recognized either the *Rosa26* locus (gRosa26-RNP) or tyrosinase locus (gTyr-RNP) was transduced into ES cells with pR26-CE as the KI donor using electroporation (Fig. 2A). The indel mutation efficiency with gTyr-RNP, via the T7 endonuclease 1 (T7E1) assay, was 90.5% in average (n = 3, 88.6%, 90.4%, or 92.5% in each replicate) (Fig. 2B). Seven to 10 days following electroporation, EGFP-positive ES cells were confirmed using microscopy (Fig. 2C) or flow cytometry (Fig. 2D). The ratio of EGFP-positive ES cells in the pR26-CE and gRosa26-RNP-transduced group was 23.8 ± 3.2% (Fig. 2E). By contrast, EGFP-positive cells rarely appeared when pR26-CE was transduced with gTyr-RNP (Fig. 2E). Next, we transduced gRosa26-RNP and CAG-EGFP plasmid flanked with (pR26-CE) or without (pCE) Rosa26-homology arm into ES cells, and the GFP expression was determined by flow cytometry 7 to 10 days following electroporation (Fig. 2F). The ratio of EGFP-positive ES cells in the pR26-CE and the gRosa26-RNP-transduced group was 19.2 ± 1.2%, whereas EGFP-positive cells rarely appeared (0.68 ± 0.1%) when homologous arm-less pCE was transduced with gRosa26-RNP (Fig. 2G,H). These results suggested that circular plasmid DNA is integrated into the genome by homology arm-mediated homologous recombination. *Rosa26* is known as stable open chromatin in virtually all tissues, including ES cells; thus, integration efficiency is thought to be relatively higher than that of other genomic loci in mice^28^. We next investigated whether this CRISPR/Cas9-mediated plasmid KI without drug selection worked efficiently in various loci in mouse ES cells. First, we targeted the promoter-less T2A-mCherry cassette to the C-terminus of the *Nanog* gene, of which expression is high in ES cells. This targeting vector has no exogenous promoter, so the fluorescent signal of mCherry can be observed only when the T2A-mCherry cassette is correctly KI in the *Nanog* locus by the endogenous promoter activity (Supplementary Fig. S4A). Transduction of pNanog-T2A-mCherry (pNmC) and gNanog-RNP into ES cells resulted in 37.5% (18/48) of ES cell clones showing mCherry signal (Supplementary Fig. S4B). We randomly selected eight mCherry-positive clones and analyzed KI by PCR. As a result, all analyzed clones were confirmed to be KI of mCherry at the *Nanog* locus (Supplementary Fig. S4C). We next targeted nine independent loci on six different chromosomes using various ES cell lines from C57BL/6J (B6J), C57BL/6N (B6N), BALB/c, or B6-129 F1 backgrounds and successfully developed precise KI in all nine attempted loci. The KI ratio ranged from 6.8 to 59.1% (Table 1). The expression levels of each endogenous gene at the KI loci in ES cells were analyzed using a public database (https://www.ebi.ac.uk/gxa/experiments/E-GEOD-27843/Results) and shown in Table 1. Every ES cell line used in this study could contribute to chimeric offspring following blastocyst injection and embryo transfer to a surrogate mother (Supplementary Fig. S5). These results suggest that drug selection-free plasmid KI method is applicable in a range of various loci in ES cells.

**Table 1:** gRNA-Cas9 RNP mediated DNA fragment KI effectively works on various genomic loci.

ID	Chromosome	ES cell strain	5' HA length (bp)	3' HA length (bp)	Insert size (bp)	Number of analyze colonies	Number of KI colonies	Ratio (%)	KI cassette	Endogenous transcript expression levels of KI locus gene in ESC*	Chimera mice development	Germ line transmission
R1-A	Chr7	B6J	997	775	1655	31	8	25.8	human gene CDS	ND	Yes	Not yet
R2-10	Chr7	B6N	997	1288	2058	22	4	18.2	Cre-T2A-EGFP-pA	3	Yes	Yes
R2-11	Chr17	B6N	300	50	1035	22	2	9.1	human gene CDS	ND	Yes	Yes
R2-16	Chr1	B6J	1000	1000	1749	22	3	13.6	DsRedexpess2-T2A-rtTA3-pA	111	Yes	Not yet
R2-18	Chr4	B6J	997	1000	2569	24	3	12.5	CreERT2-pA	3	Yes	Not yet
R2-25	Chr17	B6J	1000	1000	3788	22	13	59.1	CreERT2-T2A-tdTomoto-pA	ND	Yes	Yes
R2-A	Chr7	BALB/c	983	931	651	22	3	13.6	human gene CDS	19	Yes	Yes
R2-B	Chr11	BALB/c	650	935	654	44	3	6.8	human gene CDS	ND	Yes	Yes
R2-C	Chr14	B6-129 F1	1083	810	2558	22	9	40.9	T2A-ZsGreen1-pA	13	Yes	Yes
R4-3	Chr6	B6-129 F1	1204	3526	11,741	41	5	12.2	rtTA-pA-tetO-GOI1-T2A-GOI2-pA-promoter-GOI3	14	Yes	Not yet
										(Rosa26 = 14, Nanog = 389, Pou5f1 = 1154)		

*TPM were determined by using a public transcriptome database (https://www.ebi.ac.uk/gxa/experiments/E-GEOD-27843/Results) provided by EMBL-EBI.

### KI efficiency increased significantly with a targeting vector carrying drug-resistant gene cassettes

**Figure 3:**
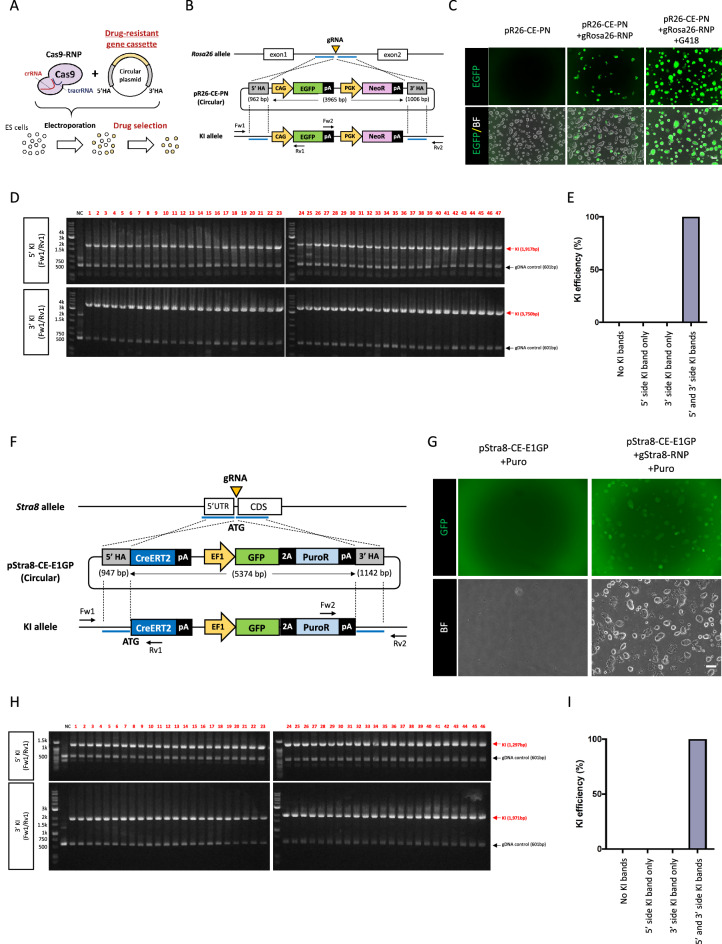
KI efficiency increased drastically with targeting vector carrying a drug-resistant gene cassette. (**A,B**) Schematic representation of *Rosa26* locus KI and drug selection strategy. (**C**) Representative ES cell colonies under a fluorescent microscope. Top panels show EGFP images and bottom panels show merged images of EGFP and bright field. Left: no electroporation (No-EP); middle: transduction of targeting vector with gRosa26-RNP without drug selection; right: transduction of targeting vector with gRosa26-RNP followed by G418 selection. Scale bar, 50 µm. (**D,E**) Genomic PCR analyses of ES cell clones. Gel images or KI ratios are shown in D or E, respectively. Red arrows in the upper or bottom panels indicate the 5′ or 3′ *Rosa26* KI, respectively, Black arrows indicated genomic DNA (gDNA) PCR control. Both 5′ and 3′ PCR positive clones are indicated with red numbers. NC: negative control using genomic DNA from wild-type B6 mouse tail. (**F**) Schematic representation of *Stra8* locus KI strategy. (**G**) Representative ES cell colonies under a fluorescent microscope. The top panels show GFP images, and the bottom panels show bright field. Left: transduction of targeting vector without gStra8-RNP followed by puromycin selection; right: transduction of targeting vector with gStra8-RNP followed by puromycin selection. Scale bar, 50 µm. (**H,I**) Genomic PCR analyses of ES cell clones. Gel images or KI ratios are shown in **H** or **I**, respectively. Red arrows in the upper or bottom panels indicate the 5′ or 3′ *Stra8* KI, respectively. Black arrows indicated genomic DNA (gDNA) PCR control. Both 5′ and 3′ PCR positive clones are indicated with red numbers. *NC* negative control using genomic DNA from wild-type B6 mouse tail.

Although the ratio of precise KI clones using the drug selection-free plasmid KI method from the total was between 20 and 30% in the case of *Rosa26* targeting (Figs. 1E, 2E), the KI ratio among the EGFP-positive ES cell clones was 87.2% (41/47) (Fig. 1F,G), suggesting that the frequency of random integration of the targeting vector into the genome was relatively low. Thus, we hypothesized that KI efficiency could be drastically increased using a targeting vector that incorporated a drug resistance cassette (Fig. 3A). We targeted pR26-CE-PN, in which the PGK promoter-driven neomycin resistance cassette is subcloned downstream of the EGFP sequence in pR26-CE, into the *Rosa26* locus accompanied with gRosa26-RNP (Fig. 3B). Several ES cell clones became EGFP-positive without G418 selection, whereas almost all clones became EGFP-positive when ES cells were treated with G418 as we hypothesized (Fig. 3C). PCR genotyping of all clones selected (47 clones) was positive for both 5′ and 3′ KI PCR bands (Fig. 3D,E).

We next investigated whether drug selection could mediate the enhancement of KI efficiency in another genomic locus. We made another targeting vector carrying a promoter-less CreERT2 followed by the EF1 promoter-driven copGFP2aPuro that was expected to be a KI at the *Stra8* locus (pStra8-CE-E1GP) (Fig. 3F). Stra8 is a germ cell-specific gene, and its expression in the ES cells is considerably low (TPM = 6, TPM of well-known pluripotent genes such as Nanog is 389, or Pou5f1 is 1154, Table 1) (https://www.ebi.ac.uk/gxa/experiments/E-GEOD-27843/Results). Induction of pStra8-CE-E1GP into ES cells without Cas9-RNP cutting the *Stra8* locus (gStra8-RNP) did not produce stable ES cell clones following puromycin selection (Fig. 3G). By contrast, 17.5 ± 3.0% ES cell became GFP positive by introducing pStra8-CE-E1EP combined with gStra8-RNP without puro selection (Supplementary Fig. S6A,B), and almost all colonies showed GFP signals after puromycin selection (Fig. 3G). PCR confirmed site-specific KI, and consequently, all selected clones showed KI PCR bands for both the 5′ and 3′ sides (46 independent clones) (Fig. 3H,I). We randomly selected 11 clones and further analyzed the sequences of their KI sites. These transgenes contained the KI precisely in-frame in the *Stra8* locus (Supplementary Fig. S6C,D). These results indicated that the efficiency of CRISPR/Cas9-mediated plasmid KI could achieve a high KI ratio up to 100% in ES cells with drug selection.

### Single electroporation could achieve dual allele KI in ES cells

**Figure 4:**
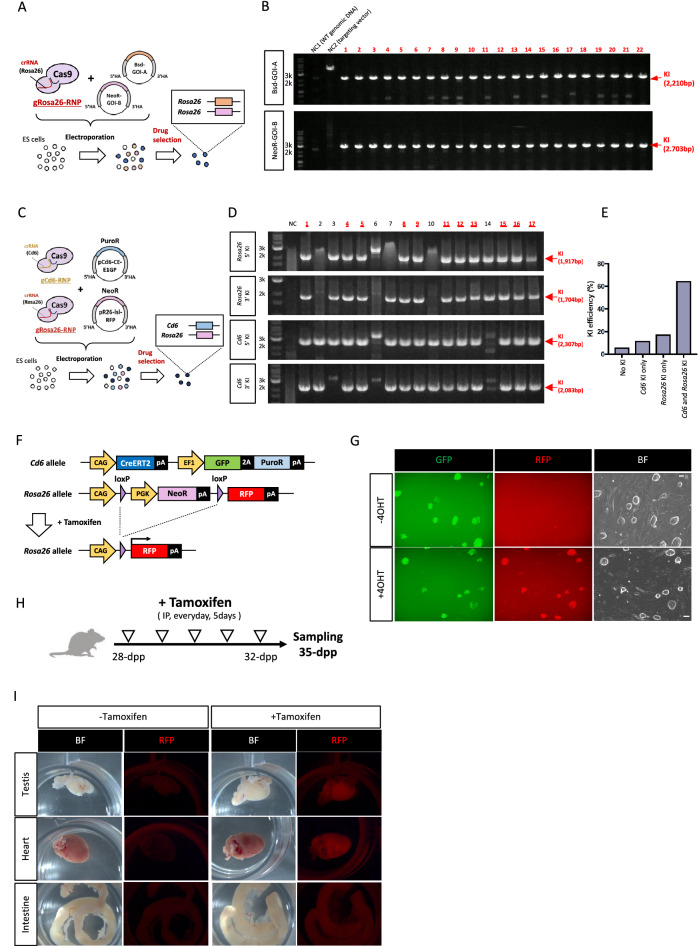
Dual allele KI in ES cells could be achieved via single electroporation. (**A**) Schematic representation of *Rosa26* locus KI strategy using two independent targeting vectors. (**B**) Genomic PCR analyses of ES cell clones. Red arrows in the upper or bottom panels indicate Bsd-GOI-A KI or NeoR-GOI-B, respectively. Black arrows indicate genomic DNA (gDNA) PCR control. Both 5′ and 3′ PCR positive clones are indicated with red numbers. NC1: negative control using genomic DNA from wild-type B6 mouse tail. NC2: negative control using the targeting vector. (**C**) Schematic representation for the combined *Rosa26* and *Cd6* locus KI strategy using two independent targeting vectors and gRNA-RNPs. (**D,E**) Genomic PCR analyses of ES cell clones. Gel images or KI ratios are shown in **D** or **E**, respectively. Red arrows indicate the 5′ or 3′ KI specific bands in *Rosa26* or *Cd6* loci, respectively. Both *Rosa26* KI and *Cd6* KI clones are indicated with red numbers. NC: negative control using genomic DNA from wild-type B6 mouse tail. (**F**) A schematic illustration of the genetic construct for tamoxifen-inducible Cre/loxP recombination in Rosa26 and *Cd6* loci. (**G**) Representative double KI ES cell colonies under a fluorescent microscope without (top panels) or with (bottom panels) 4-hydroxytamoxifen (4OHT). Left panels show GFP, middle panels show RFP, and left panels show bright field. Scale bar, 50 µm. (**H**) A schematic illustration of tamoxifen administration followed by tissue sampling in the chimeric mice having both *Rosa26* KI and *Cd6* KI alleles. (**I**) Expression of RFP in tissues of chimeric mice. Mice were administered with tamoxifen intraperitoneally 5 times and were then used for fluorescent microscope observation.

Since the KI efficiency could be increased using plasmid KI with drug selection, we hypothesized that it would be possible to KI multiple alleles simultaneously by using targeting vectors with different drug selection cassettes. To investigate this hypothesis, we first targeted two different gene cassettes into the same locus, *Rosa26*, by single electroporation (Fig. 4A). PCR genotyping following electroporation and subsequent G418 and blasticidin selection revealed that all clones selected (22 clones) contained KI for both cassettes (Fig. 4B). We then determined whether single electroporation could achieve double KI into different genomic loci. For this purpose, we targeted the CAG-CreERT2-EF1-copGFP2aPuro cassette into the *Cd6* locus (pCd6-CE-E1GP) and the CAG-loxP-Neo-loxP-Red fluorescent protein (RFP) cassette into the *Rosa26* locus (pR26-lsl-RFP) with gCd6-RNP and gRosa26-RNP into ES cells by single electroporation (Fig. 4C). The *Cd6* locus is another safe harbor locus for expecting stable gene expressions ubiquitously in various tissues in mice^35^. After G418 and puromycin selection for 7 days, we selected 17 ES cell clones for PCR analysis to determine the KI of *Cd6* and *Rosa26* loci. Strikingly, 11 out of 17 ES cell clones showed both the *Cd6* and *Rosa26* KI bands (64.7%, Fig. 4D,E). Using the double KI ES clones, we demonstrated that both KI alleles worked in vitro as RFP signals were only observed after 4OHT was administered in the culture medium (Fig. 4F,G). We further determined whether these KI alleles also worked correctly in vivo. Chimeric mice were produced by injecting the ES cell clones into preimplantation blastocyst followed by chimera embryo transfer to pseudopregnant surrogate mothers. Chimeric mice at 28 days postpartum (Supplementary Fig. S7) were intraperitoneally administered with tamoxifen once daily for 5 days, and then tissues were collected and used for microscopic analysis to evaluate RFP fluorescein expression (Fig. 4H). RFP signals were observed in various tissues from chimeric mice administered with tamoxifen (Fig. 4I), indicating that integrated transgenes worked as expected in vivo in mice.

## Discussion

We aimed to develop a simple and highly efficient KI method in mouse ES cells to efficiently develop genetically manipulated mouse models. We found that induction of circular plasmid DNA, as a targeting vector, besides KI site specific Cas9-RNP, enabled efficient DNA KI in the absence of drug selection. Furthermore, incorporating a drug-resistant gene cassette into the circular targeting vector produced multiple KI in single electroporation with extremely high efficiency. The largest size of gene cassette used in this study for KI was 11.7 kbp without drug selection and 6.2 kbp with drug selection. The length of DNA fragments frequently used for making gene-manipulated mice such as CreERT2, EGFP, or Cas9 are approximately 2.5, 1, or 4 kbp, respectively. Thus, we believe that CRISPR/Cas9-mediated plasmid KI can be universally applied in ES cells and is highly beneficial for the efficient generation of genetically modified mice.

Previous studies using mouse ES cells^36^ and fibroblasts^37^ have shown that introduction of plasmid DNA into cells resulted in very limited genome integration. These studies are consistent with our data here where a circular plasmid with homology arms was rarely incorporated into the genome of ES cells (Fig. 1C,D; Supplementary Fig. S1C,D). Hence, a linearized DNA fragment with homology arms, which is integrated into the genomic loci by homology-dependent repair mechanism, has been conventionally used for site-specific KI^3–5^. However, although linearization of plasmid DNA significantly increases the genomic integration rate, much of the integration is nonspecific even if the DNA has homologous arms^4^. Similar findings were observed in this study, i.e., all ES cell clones that became drug-resistant could not be confirmed to be accurate KI when a linearized DNA fragment with a drug-resistant gene was introduced into ES cells without CRISPR/Cas9 (Supplementary Fig. S2). On the other hand, using CRISPR/Cas9 genome editing, it has been reported that the long DNA KI efficiency is increased in mouse ES cells^38^. This method introduced the circular plasmid into ES cells, followed by linearization with CRISPR/Cas9. Nevertheless, the accurate KI ratio was about half of the gene-integrated ES cells, and the rest was incomplete partial KI or random integration^38^. Also, the efficiency of CRISPR/Cas9-mediated long DNA KI in zygote was improved when the double-strand targeting plasmid was linearized intracellularly by CRISPR/Cas9 compared to the circular one^16,39,40^, whereas no significant difference was observed between circular plasmid or linearized plasmid for KI efficiency in the case in mouse ES cells^16^. Our current results showed that circular plasmid was able to be KI in various chromosomes using CRISPR/Cas9-RNP even without drug selections, and the ratio was up to around 60%. Therefore, at least in the case of KI to ES cells, it would be said that sufficiently high KI efficiency can be achieved by using Cas9-RNP and circular plasmid as the targeting vector.

KI efficiency in ES cells was significantly higher in Cas9-RNP than Cas9 plasmid in this study. Unlike Cas9-plasmid, which requires intracellular transcription and translation, Cas9-RNPs rapidly enter the nucleus and cleave the genome after transfection. Previous reports have shown that Cas9-RNPs significantly increase genome editing efficiency compared to Cas9-plasmids^41,42^. Therefore, it is suggested that the increased rate of genomic double-strand breaks by Cas9-RNPs led to the increased KI efficiency in ES cells observed in this study.

It is also notable that Cas9-RNPs are degraded more quickly in the cell than Cas9-plasmids, and thus off-target cleavage occurs at a lower rate^41^. Furthermore, high-fidelity (HiFi) type Cas9 protein was used as an RNP component in this study. Several papers have reported that off-target cleavage of HiFi type Cas9 is remarkably lowered, as low as un-detectable levels by analyzing a next-generation sequencer^43,44^. Thus, the present KI method using HiFi Cas9 protein would also consider site-specific, although further analysis is needed to clarify the presence or absence of off-targets. On the other hand, there were cases in which the PCR KI band could be observed only on either the 5' side or the 3' side in this experiment, suggesting that incomplete KI occurred by this method to some extent. Therefore, it is essential for KI screening to perform both 5' side and 3' side PCRs. It is also noted that the 5' or 3' sides may be KI to different chromosomes; thus, confirming the KI by PCR using primers designed outside each HAs to amplify the entire targeted allele would also be helpful.

Additionally, simultaneous gene KI in multiple loci was achieved with high efficiency by using plasmids with different drug resistance gene cassettes as targeting vectors (Fig. 4). This would theoretically halve the duration for developing dual gene-manipulated ES cell clones compared with the sequential manipulations of two genes. Extended culture of ES cells is known to lead to a more aberrant methylation status of DNA and an increase in chromosomal instability^25,27^, resulting in reduced pluripotency^25^. Thus, modifying multiple genes in single electroporation with a shorter culture period would be advantageous for developing chimeric mice while maintaining the pluripotent quality of ES cells.

Generally, mice with double mutations have been developed by mating with each mutation, and consequently obtaining age-suitable individuals for phenotypic analysis can take several months. Present plasmid KI method with drug selections would enable the manipulation of multiple genes in chimeric mice in a short period and thus would be remarkably useful especially in fields where phenotypic analysis could be performed in chimeric animals^45^.

The contribution of blastocyst-injected ES cells to the germ cells, i.e., sperm or oocyte, in a chimera (germline transmission, GLT), is a significant issue in producing stable genetically manipulated mice via chimeric mice^46,47^. Particularly, chimeric mice produced using ES cells derived from inbred lines such as C57BL/6 and BALB/c frequently have poor GLT^46^. GLT can be variable and inefficient, partly because there is competition between the host and donor cells in chimeras that is affected by the quality of ES cells^46^. Several recent studies suggested that the blastocyst complementation method, whereby ES cells were injected into genetically germ cell-less blastocysts, can significantly improve the efficiency of GLT^46,47^. Although six mouse lines out of nine attempts achieved GLT in our present experiment (Table 1), it would be worthwhile, in future investigations, to combine our gene targeting technology with blastocyst complementation methods to establish more efficient technologies for producing genetically modified animals that can consistently achieve GLT.

An additional point of concern is backcrossing. In certain research areas, analyses using a specific pure-strain mouse are required. Thus, if ES cell-based chimeric mice or zygote genome-edited mice are generated using undesired strain, it is necessary to backcross 6 to 10 times to obtain a congenic strain before phenotypic analysis is carried out. This process requires a long time, more than a year. In this experiment, we demonstrated that accurate and highly efficient gene targeting is possible using ES cells from various genetic backgrounds, such as hybrids of the B6-129 F1 line, or pure lines of B6N and BALB/c. The injection of those ES cells into 4n tetraploid blastocyst would enable in vivo gene function analysis using pure inbred mouse lines in the F0 generation. Future works will require the establishment of high-quality ES cells from additional pure mouse lines to avoid the time-consuming backcrossing.

In conclusion, our study demonstrates that multiple KI edits can be made with high efficiency in a single step using a circular plasmid with Cas9/RNP genome editing in murine ES cells. We believe that this technique could help to substantially reduce both the amount of time taken in the conventional production of genetically modified chimeric mouse models and the number of animals currently used in this process.

## Materials and methods

### Animals and ethics

C57BL/6J or BALB/c mice were purchased from Clea-Japan (Tokyo, Japan), and ICR mice were purchased from Japan SLC (Shizuoka, Japan) or Clea-Japan. Mice were housed in a pathogen-free condition under a 12 h light/12 h dark photoperiodic cycle with food and water ad libitum in the experimental animal facility at the Institute of Medical Science, University of Tokyo. All mouse experiments were approved by the Institutional Animal Care and Use Committee of the University of Tokyo (approval number PA17-63) and performed according to their guidelines as well as the ARRIVE guidelines (https://arriveguidelines.org).

### Mouse embryonic fibroblast propagation and ES cell culture

To obtain mouse embryonic fibroblasts (MEFs), BALB/c and C57BL/6J mice were mated, and pregnant females were euthanized by cervical dislocation on E14.5. MEFs were isolated by homogenizing collected fetuses and then by culturing these cells in Dulbecco’s modified Eagle medium (DMEM) (Nacalai, Kyoto, Japan) containing 10% (v/v) fetal bovine serum (FBS) (Sigma-Aldrich, St. Louis, MO, USA), 100 U/mL penicillin and 100 µg/mL streptomycin (FUJIFILM Wako Pure Chemical, Osaka, Japan) at 37 °C in humidified 5% CO_2_. After 12–14 days of culture, proliferating MEFs were irradiated with X-rays (50 Gy) to halt the cell cycle, and the mitotically inactivated MEFs were then used for ES cell culture for the feeder culture condition. ES cells having C57BL/6J or BALB/c background were developed in our laboratory^25^, with slight modifications. In brief, mated females were euthanized by cervical dislocation, and embryos were collected by flushing the oviduct a day after observation of the virginal plug. Collected embryos were cultured for 2 days in potassium simplex optimized medium (KSOM) (Merck-Millipore, Darmstadt, Germany) to produce blastocysts. Each blastocyst was transferred to a well in a 24-well culture plate (BD Biosciences, Bedford, MA, USA) containing irradiated MEFs (1.2 × 10^5^/cm^2^) and cultured in ES culture medium (ESCM; Knockout-DMEM; Thermo Fisher Scientific, Waltham, MA, USA) with 15% (v/v) FBS (Thermo Fisher Scientific), 2 mM GlutaMax (Thermo Fisher), 100 U/mL penicillin (FUJIFILM Wako Pure Chemical), 100 µg/mL streptomycin (FUJIFILM Wako Pure Chemical), 0.1 mM 2-mercaptoethanol (Thermo Fisher Scientific), leukemia inhibitory factor and t2i (0.2 µM PD0325901 (Sigma-Aldrich), and 3 µM CHIR99021 (Axon Medchem, Groningen, Netherlands)). Five to 7 days after the blastocyst seeding, inner cell mass outgrowth was digested into single cells by treatment with 0.25% (w/v) trypsin-ethylenediaminetetraacetic acid (EDTA) (Nacalai) and then passaged onto fresh MEFs and cultured in ESCM for developing self-renewing ES cells. Besides these in-house developed ES cell lines, JM8.A3 (C57BL/6N)^48^ or V6.5 (B6-129 F1) ES cell lines were also used in experiments. Each ES cell line was passaged every 2–3 days at a subculture dilution of 1:10. For feeder-free ES cell culture, dishes precoated with 0.1% (w/v) gelatin solution (Sigma-Aldrich) were used, and ES cells were cultured in ESCM.

### Plasmids

A pAAV-mRosa26-CAG-EFGP (pR26-CE) targeting vector with 5′ (962 bp) and 3′ (1,006 bp) homology arms to *Rosa26* or pAAV-CAG-EGFP (pCE) have been previously reported^20^ and was kindly gifted from Dr. Mizuno. To make pRosa26-CAG-EGFP-PGK-NeoR plasmid (pR26-CE-PN), the DNA fragment coding PGK-NeoR-bGHpA was PCR amplified using pL452 as a template and cloned between EGFP and the 3′ homology arm sequences of pR26-CE using an In-Fusion HD Cloning Kit (Takara, Shiga, Japan). pNanog-T2A-mCherry (pNmC) or pStra8-CreERT2-EF1-copGFP2aPuro (pStra8-CE-E1GP) was constructed using pUC19 as a backbone. For making pNmC, the 5′ and 3′ homology arms of the *Nanog* locus were PCR amplified using C57BL/6 J genome as templates. Each homology arm and T2A-mCherry-bGHpA was cloned into pUC19 using the In-Fusion HD Cloning Kit (Takara). For making pStra8-CE-E1GP, DNA coding CreERT2-Rabbit globin poly-A was purchased from Genewiz (Genewiz Japan, Saitama, Japan). The 5′ and 3′ homology arms of the *Stra8* locus and EF1-copGFP2aPuro DNA fragments were PCR amplified using C57BL/6 J genome or PB513 (System Biosciences, Palo Alto, CA, USA) as templates, respectively. Each fragment was cloned into pUC19 using the In-Fusion HD Cloning Kit (Takara). To make pAAV-mRosa26-CAG-loxP-Neo-loxP-RFP (pR26-lsl-RFP), the loxP-Neo-loxP fragment or RFP fragment was PCR amplified using pROSA26-DEST (#21189, Addgene) or PB514 (System Biosciences) as templates, respectively, and cloned in between the CAG promoter and 3′ homology arm of pR26-CE using the In-Fusion HD Cloning Kit (Takara). To make pCd6-CAG-CreERT2-EF1-copGFP2aPuro (pCd6-CE-E1GP), homology arms for the *Cd6* locus or EF1-copGFP2aPuro fragments were PCR amplified using genomic DNA or PB514 (System Biosciences) as templates, respectively, whereas the CreERT2 fragment was purchased from Genewiz (Genewiz Japan). All the DNA fragments were cloned into pAAV-MCS2 (#46954, Addgene) using the In-Fusion HD Cloning Kit (Takara) to make pCd6-CE-E1GP. An all-in-one plasmid expressing Cas9 mRNA and guide RNA (gRNA), which targets a specific gene of interest were prepared by cloning double-stranded oligos into the BbsI site of pX459 (Addgene, #48139). The gRNA targets (each 20 nucleotides long) were as follows: *Rosa26*, 5′-AAGGGATTCTCCCAGGCCCA-3′; tyrosinase, 5′-GGTCATCCACCCCTTTGAAG-3′; *Nanog*, 5’-TATGAGACTTACGCAACATC-3’; *Stra8*, 5′-TAGATTATAATGGCCACCCC-3′; *Cd6*, 5′-ACAAGTTGGGAAAGGTTTAT-3′. A summary of the targeting vectors used in this study is shown in Supplementary Table S1. Detailed targeting vector information or gRNA sequences for other target loci, assigned with unique project accession numbers (R1-A, R2-10, R2-11, R2-16, R2-18, R2-19, R2-A, R2-B, and R2-C in Table 1 or Bsd-GOI-A and NeoR-GOI-B in Fig. 4) will be reported in a separate publication along with their corresponding mouse phenotypes.

### Preparation of CRISPR/Cas9 ribonucleoprotein complex (RNP) and electroporation of ES cells

TracrRNA, crRNA, and Cas9 protein were purchased from IDT (Coralville, IA, USA). TracrRNA and crRNA were dissolved in Duplex Buffer (IDT, 200 µM each) and annealed in a thermal cycler at 95 °C for 10 min followed by − 1 °C/min stepdown cycles until 25 °C. Annealed RNA (100 µM) were then incubated with 3 µg/µL Cas9 protein at 37 °C for 20 min to form Cas9-RNP. A Neon Transfection System (MPK5000, ThermoFisher) was used for electroporation of plasmid and Cas9-RNP into ES cells. In brief, 1 × 10^5^ ES cells with 1 µg of targeting vector were electroporated with or without all-in-one CRISPR/Cas9 vector (0.5 µg) or Cas9-RNP (at a final concentration of 10 µM annealed RNA and 0.3 µg/µL Cas9 protein) in a 10 µL tip (MPK1096, Thermo). The Neon system used two pulses at 1200 V and 20 ms or a single pulse at 1400 V and 30 ms for all-in-one vector transfection or Cas9-RNP transfection, respectively. Electroporated ES cells were cultured in ESCM with or without MEFs.

### Drug selection and in vitro induction of tamoxifen-mediated Cre-loxP recombination

For drug selection using either puromycin- or blasticidin-resistant genes, electroporated ES cells were treated with G418 (400 ng/mL), puromycin (0.5 µg/mL), and/or blasticidin (20 µg/mL) to develop stable transfectants. For tamoxifen-mediated in vitro Cre/loxP recombination, ES cell clones were treated with 4-hydroxytamoxifen (4OHT, 1 µM) for 2 days; fluorescent reporter expression was observed using a fluorescein microscope (BZ-X710, Keyence).

### Flow cytometry

For cytometric analysis to detect fluorescence reporter expression, ES cell colonies were digested into single cells with 0.25% (w/v) trypsin–EDTA, and the single cells were resuspended in PBS, 2 mM EDTA, and 1% (w/v) BSA. Fluorescence expression was observed using the FACSCalibur system (BD Biosciences, San Jose, CA, USA); collected data were analyzed with FlowJo software (BD Biosciences).

### Genotyping and indel mutation rate determination

Genotypes of KI in each ES cell clone were determined through PCR using genomic DNA as a template. Single ES cell colonies as clones were manually selected and passed onto a new culture dish. The expanded ES cell clone was then harvested and lysed using Tail Lysis Buffer (Nacalai) containing 7 U/mL proteinase K (#9034, Takara) at 65 °C for at least 2 h. Genomic DNA from the crude lysate was extracted and purified by conventional phenol/chloroform/isoamyl alcohol (all from Nacalai) treatment followed by ethanol precipitation for PCR analysis. KODOne polymerase (Toyobo, Osaka, Japan) was used for DNA fragment amplification. Indel mutation ratio was determined via T7 endonuclease I mismatch cleavage assay using the Alt-R Genome Editing Detection Kit (IDT). The ratio of indel mutation was calculated as described (http://crispr.technology/resources/quantification.html). Supplementary Table S2 shows the primer sequences used for KI genotyping. The details of other target loci with unique project accession numbers (R1-A, R2-10, R2-11, R2-16, R2-18, R2-19, R2-A, R2-B, and R2-C in Table 1 or Bsd-GOI-A and NeoR-GOI-B in Fig. 4) will be reported in a separate publication as mentioned above. All the raw data of agarose gel electroporation were shown in Supplementary Figs. S8 and S9.

### Chimeric mouse production and tamoxifen administration

Chimeric mice were developed by blastocyst injection of gene-targeted ES cells. In brief, 8-week-old female mice were superovulated via sequential intraperitoneal administration of 7.5 U equine chorionic gonadotropin (Serotropin, ASKA Animal Health, Tokyo, Japan) and 7.5 U human chorionic gonadotropin (Gonatropin, ASKA Animal Health) 48 h apart and then mated with a mature male of the same strain to obtain embryos. Mated females were euthanized on the next day of vaginal plug observation, and two-cell embryos were collected by perfusion of oviduct with modified Whitten medium (mWM). Collected embryos were cultured in KSOM (Merck-Millipore) for 2 days to develop blastocysts. Six to ten ES cells were injected into a single blastocyst, and the injected blastocysts were transferred into the uterus of pseudopregnant ICR female surrogates (20 to 25 blastocysts per individual female) at 2.5 day postcoitum to obtain chimeric offspring. For developing chimeras using B6J (black hair), B6N (JM8.A3, Agouti hair), or B6-129 F1 (V6.5, Agouti hair), blastocysts having an ICR background (albino) were used. In the case of BALB/c (albino) ES cell injection, blastocysts having B6J background (black hair) were used. For tamoxifen-induced Cre/loxP recombination in vivo, 5-week-old chimeric mice were intraperitoneally injected with tamoxifen (Sigma-Aldrich, 2 mg/body diluted in corn oil) or corn oil alone (control) once daily for 5 times and harvested for tissue sampling 3 days after the final tamoxifen administrations. Collected tissues were observed for fluorescent expression analysis using a fluorescent stereomicroscope (Leica Microsystems, Tokyo, Japan).

### Statistical analysis

All numerical data are shown as the mean ± SEM of three independent replications. Differences between treatments or genotypes were tested using the two-sided Student’s *t* test. P-values of less than 0.05 were considered significant.

## Supplementary Information

Supplementary Information.

## Data Availability

All research data are available in the paper and supplementary material. The datasets of vector sequences and cell lines generated during the present research are available from the corresponding author on reasonable request.
