# Supplementary material for: Efficient simultaneous double DNA knock-in in murine embryonic stem cells by CRISPR/Cas9 ribonucleoprotein-mediated circular plasmid targeting for generating gene-manipulated mice
Source: Sci Rep. 2022 Dec 13;12:21558. doi: 10.1038/s41598-022-26107-z (PMC9748034; doi:10.1038/s41598-022-26107-z)
Supplement: Supplementary file 1 — Supplementary Information. [file 41598_2022_26107_MOESM1_ESM.pdf]

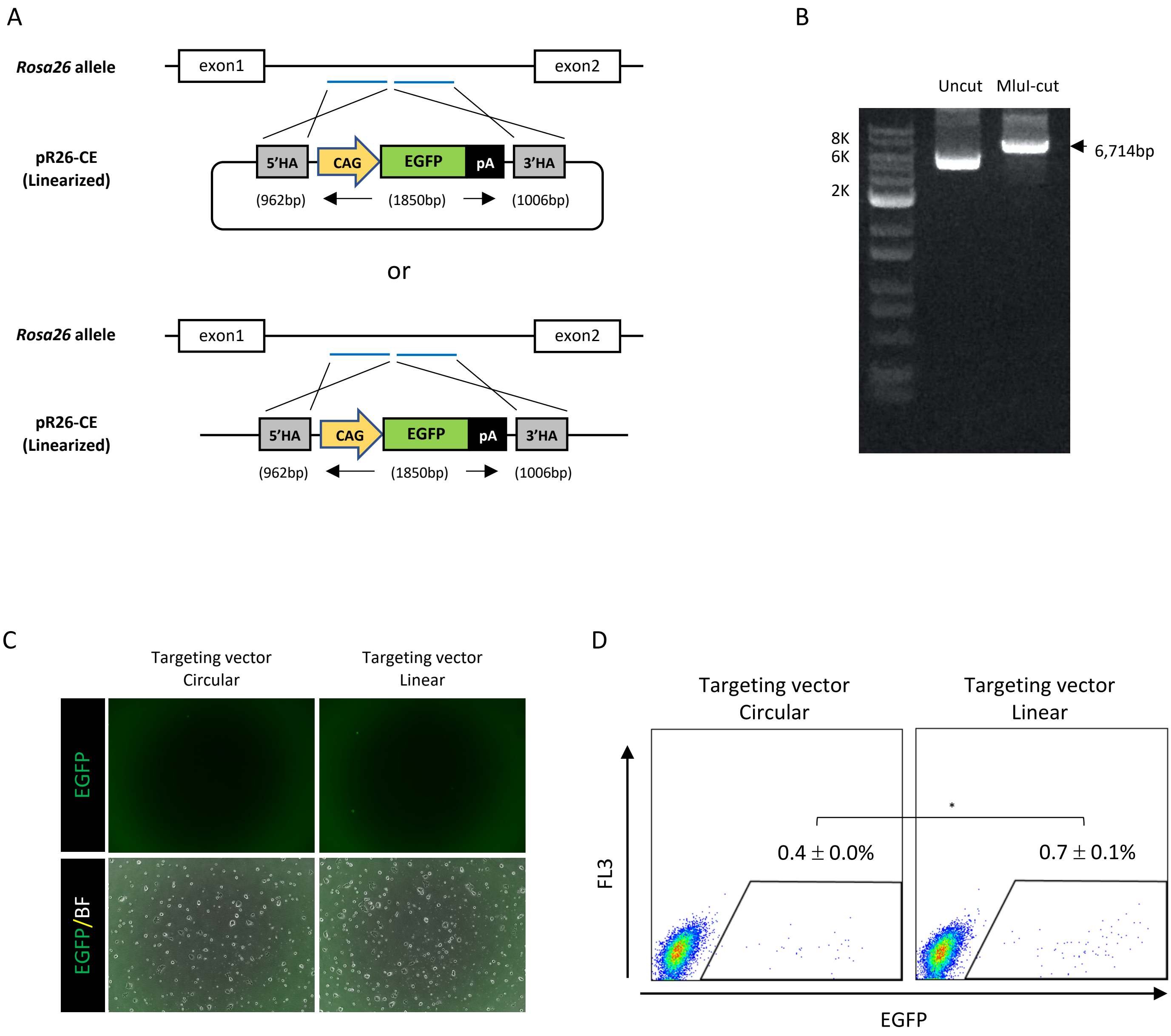

Supplemental Figure S1. *Comparison between circular and linearized plasmid DNA on genomic integration in ES cells*
(A) Schematic representation of circular or linearized GFP cassette introduction strategies. Each vector contained a CAG-EGF cassette flanked by approximately 1 kbp homology arms and was transduced into ES cells via electroporation.
(B) Electroporation band representing uncut circular targeting vector or MluI-cut linearized vector. Original gel is presented in Supplementary Figure S8.
(C) Representative ES cell colonies under a fluorescent microscope. Top panels show EGFP images and bottom panels show merged images of EGFP and bright field. Left: introduction of circular vector into ES cells; right: introduction of linearized vector into ES cells. Scale bar, 50 μm.
(D) Flow cytometry of ES cells. Gate represents EGFP-positive fraction, and the GFP positive cell ratios are shown as mean ± SEM. The asterisk depicts a significant difference (n = 3, $P < 0.05$).

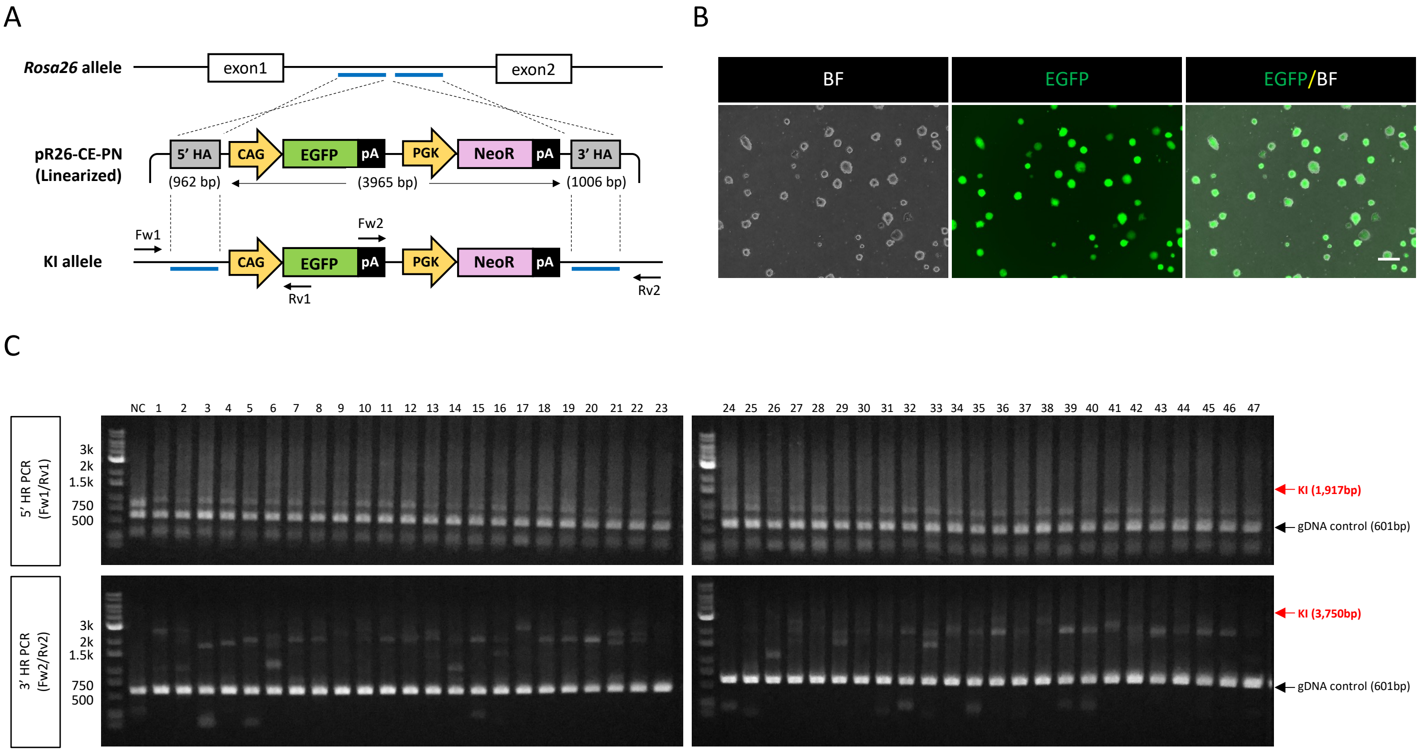

Supplemental Figure S2. *Integration of linearized DNA fragment occurs mainly random into ES cell genome*
(A) Schematic representation of linearized EGFP/neomycin cassette introduction strategies. Linearized vector containing CAG-EGFP-PGK-NeoR cassette flanked by the 5′ and 3′ homology arms (962 and1,006 bp, respectively) was transduced into ES cells via electroporation.
(B) Representative ES cell colonies under a fluorescent microscope. Linearized pR26-CE-Pn was transduced into ES cells followed by neomycin selection. Left: bright field image; middle: EGFP image; right: merged with bright field and EGFP. Scale bar, 50 μm.
(C) Genomic PCR analyses of neomycin-resistant ES cell clones for determining locus-specific cassette KI. Red or black arrows indicate the Rosa26 locus-specific KI band or genomic DNA (gDNA) PCR control band, respectively. NC: negative control using genomic DNA from wild-type B6 mouse tissue. Original gels are presented in Supplementary Figure S8.

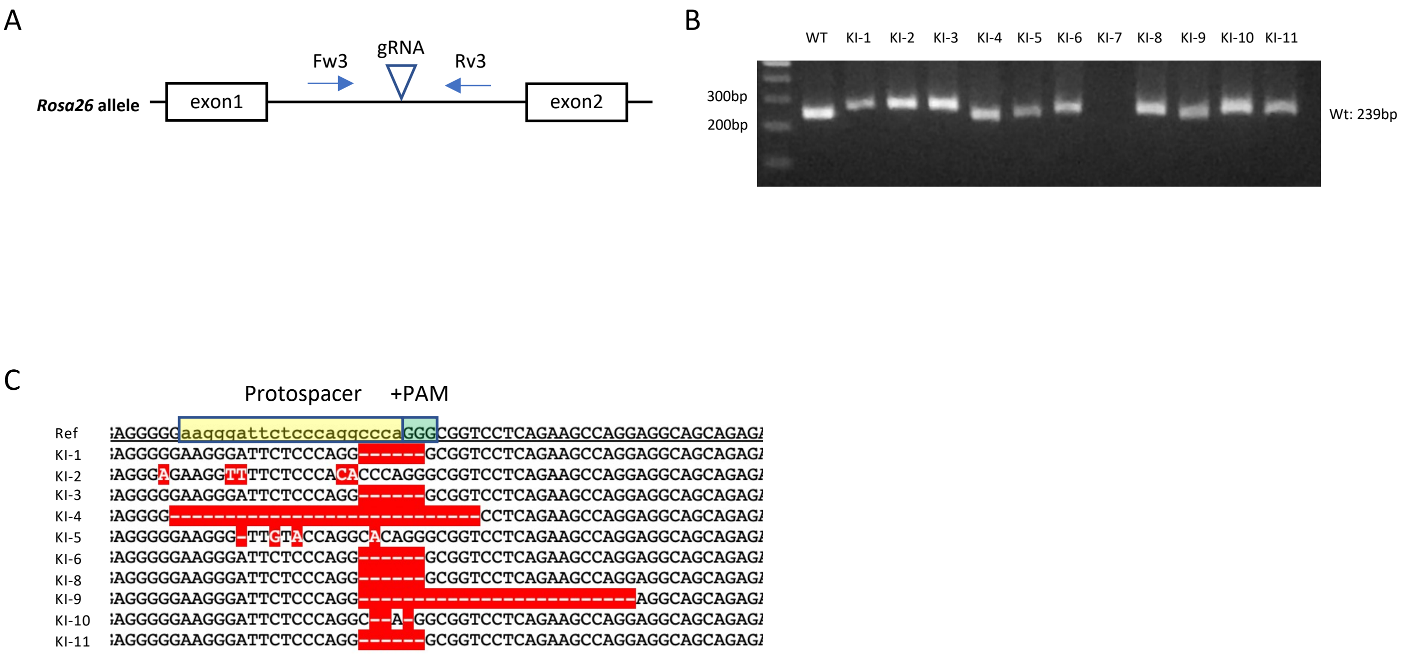

Supplemental Figure S3. *Introduction of indel mutations is frequently observed in the chromosome paired with the knock-in chromosomes.*
(A) A schematic illustration of guide RNA target position as well as 5′ and 3′ homology arms in *Rosa26* locus. Fw3: position representing forward primer. Rv3: position representing reverse primer.
(B) Genomic PCR analyses of ES cell clones using Fw3 and Rv3 primer set. Original gel is presented in Supplementary Figure S8.
(C) Sanger sequencing of genomic DNA at the *Rosa26* locus in each ES cell clone.

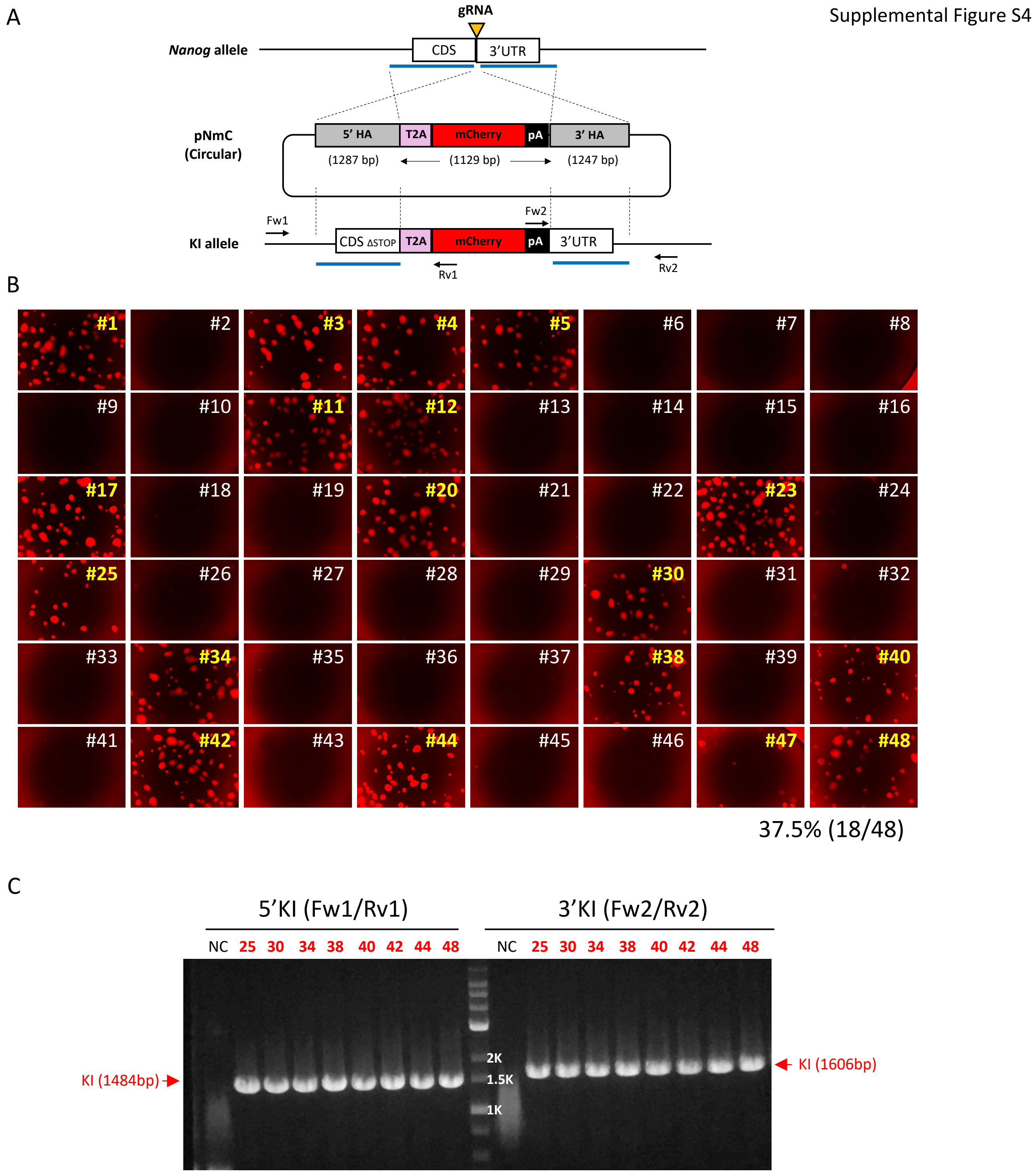

Supplemental Figure S4. *Targeting of promoter-less T2A-mCherry gene cassette into Nanog locus.*

(A) Schematic representation of circular T2A-mCherry cassette introduction strategies. Circular vector containing T2A-mCherry cassette flanked by the 5′and 3′homology arms (1287 and1247 bp, respectively) of Nanog was transduced with gNanog-RNP into ES cells via electroporation.

(B) Individual ES cell clones under a fluorescent microscope. 18 out of 48 (37.5%) picked clones showed mCherry signals.

(C) Genomic PCR analyses of the mCherry positive ES cell clones. Red arrows on the left or right side of the image indicate the 5′or 3′T2A-mCherry KI into the *Nanog* locus, respectively, Both 5′and 3′PCR positive clones are indicated with red numbers. NC: negative control using genomic DNA from wild-type B6 mouse tail.

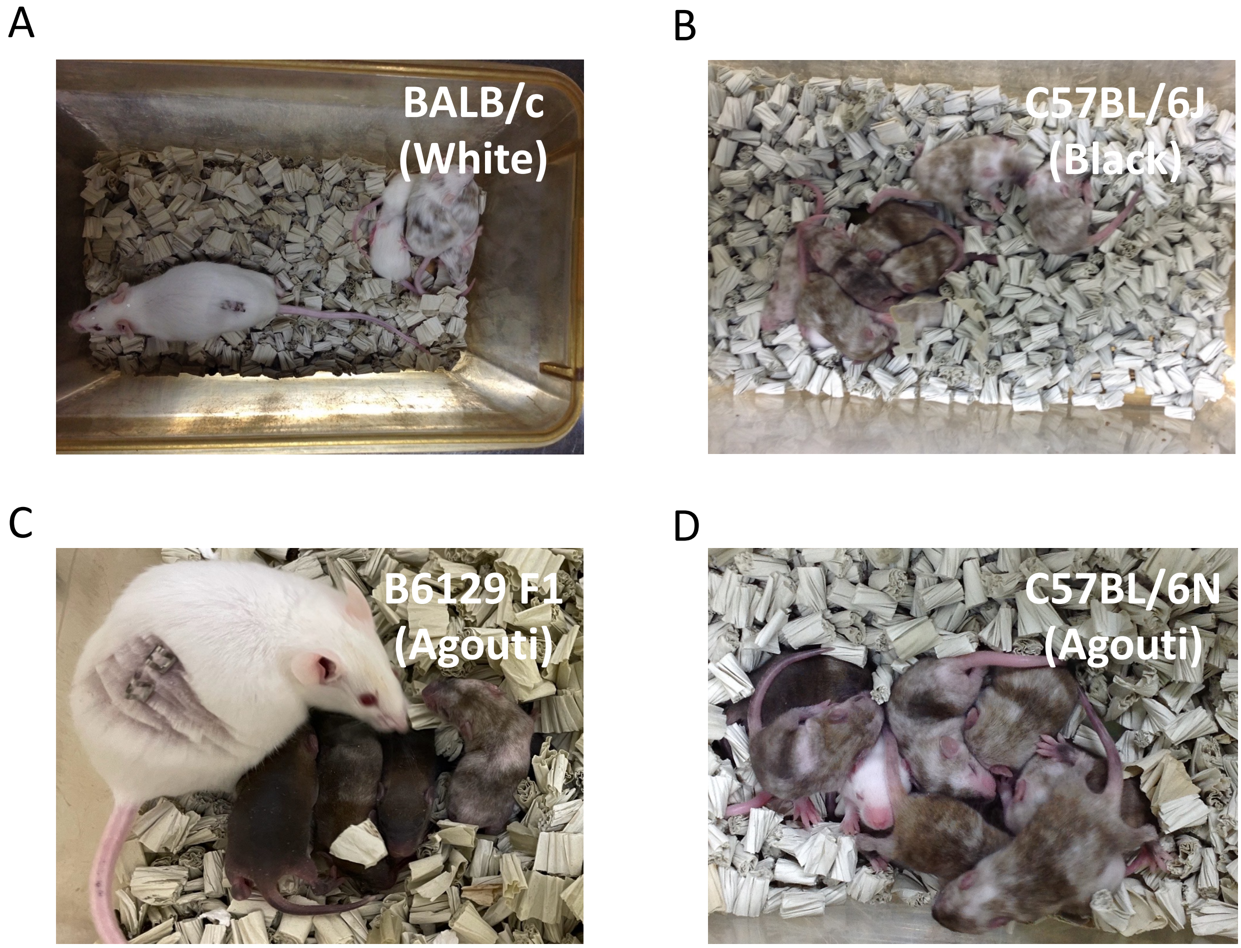

Supplemental Figure S5. *Representative images of chimera mice*.
(A) ES cells derived from BALB/c embryo (white hair) were injected into C57BL/6J (black hair) blastocysts followed by embryo transfer to mother surrogates.
(B–D) ES cells derived from C57BL/6J (black hair) (B), B6129 F1 (agouti hair) (C), or JM8.A3 ES cell line (C57BL/6N, agouti hair) (D) were injected into ICR (white hair) blastocysts followed by embryo transfer to mother surrogates.

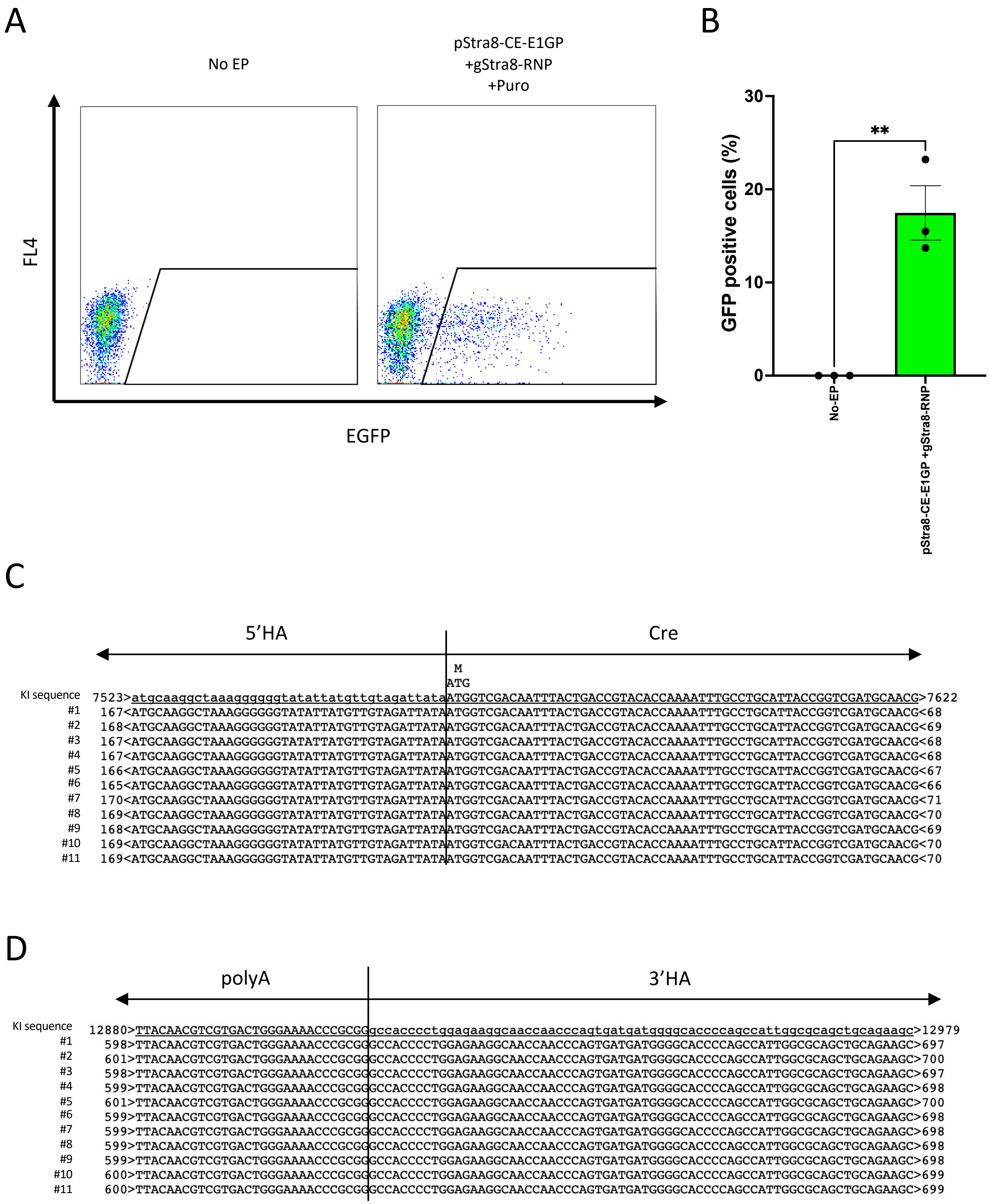

Supplemental Figure S5. *Sanger sequencing of genomic DNA at the Stra8 locus in each ES cell clone showing precise KI of gene cassette.*

(A) Flow cytometry of ES cells. Gate represents GFP-positive fraction.

(B) GFP positive cell ratios are shown as mean ± SEM. The asterisk depicts a significant difference (n = 3, P < 0.01).

(C, D) Sequence data representing 5′ (C) or 3′(D) KI at the Stra8 locus.

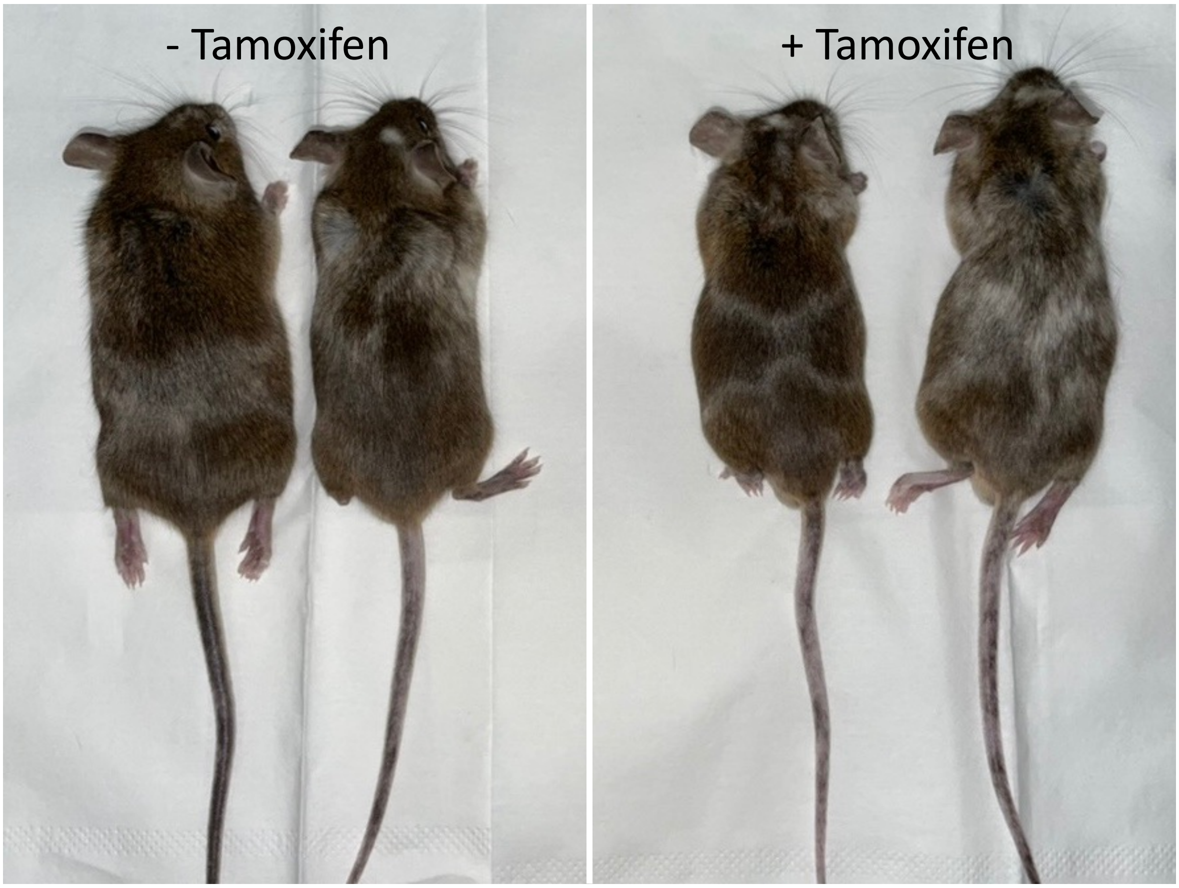

Supplemental Figure S6. *Representative pictures of chimeric mice administered with or without tamoxifen.*
B6129 F1 ES cells having agouti color gene was injected into ICR blastocyst (white hair) for developing the chimera. Chimerism determined by coat color in each chimera was more than 90%.

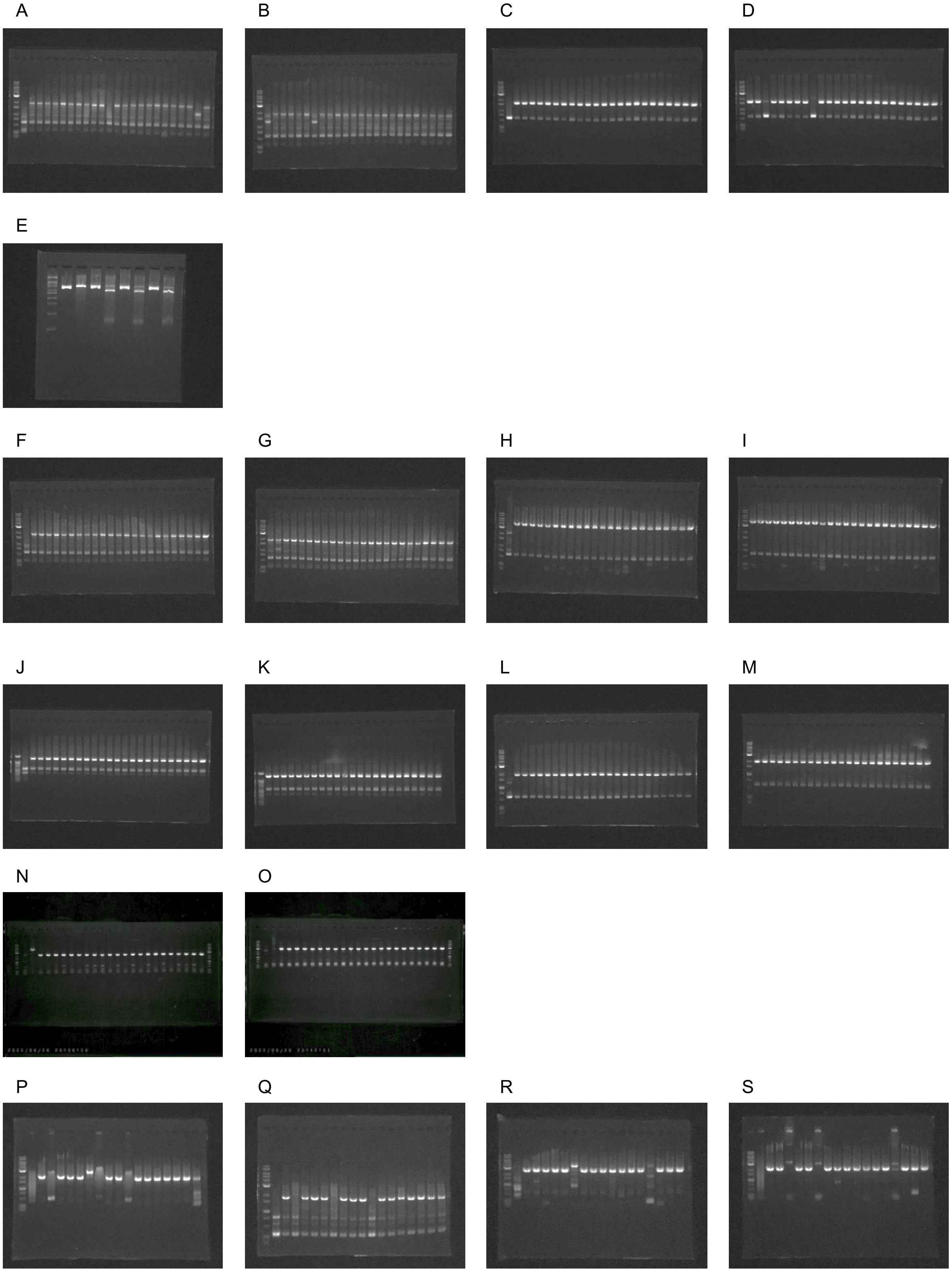

Supplemental Figure S7. *Original gel images of DNA electrophoresis presented in the manuscript.*
(A-D) Original gel images presented in Figure 1F, top left (A), top right (B), bottom left (C), or bottom right (D).
(E) Original gel image presented in Figure 2B
(F-I) Original gel images presented in Figure 3D, top left (F), top right (G), bottom left (H), or bottom right (I).
(J-M) Original gel images presented in Figure 3H, top left (J), top right (K), bottom left (L), or bottom right (M).
(N, O) Original gel images presented in Figure 4B, top (N), or bottom (O).
(P-S) Original gel images presented in Figure 4D, 1st top (P), 2nd top (Q), 3rd top (R), or bottom (S).

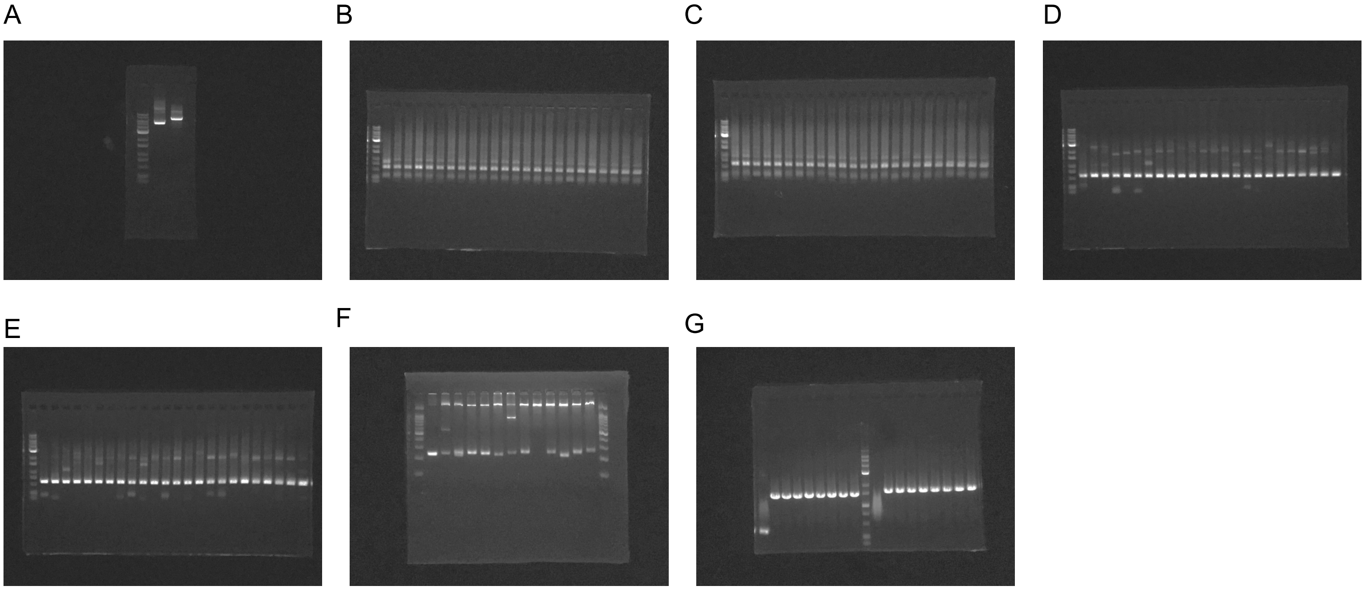

Supplemental Figure S8. *Original gel images of DNA electrophoresis presented in the Supplemental Figures.*
Original gel images of DNA electrophoresis presented in the Supplemental Figures.
(A) Original gel images presented in Supplemental Figure S1B.
(B-E) Original gel images presented in Supplemental Figure S2C, top left (B), top right (C), bottom left (D), or bottom right (E).
(F) Original gel images presented in Supplemental Figure S3B.
(G) Original gel images presented in Supplemental Figure S4C.

Summary of targeting vectors used in this study

Name of targeting vector	Locus (Chromosome)	KI gene	KI cassette size (bp)	5' HA (bp)	3' HA (bp)	Selection casette
pR26-CE	Rosa26 (6)	CAG-EGFP	1,850	962	1,006	no
pR26-CE-PN	Rosa26 (6)	CAG-EGFP-PGK-NeoR	3,965	962	1,006	Puromycin
pNmC	Nanog (6)	T2A-mCherry	1,129	1,287	1,247	no
pStra8-CE-E1GP	Stra8 (6)	CreERT2-EF1-copGFP2aPuro	5,347	947	1,142	Puromycin
pR26-lsl-RFP	Rosa26 (6)	CAG-loxP-Neo-loxP-RFP	4,839	962	1,006	Neomycin
pCd6-CE-E1GP	Cd6 (19)	CAG-CreERT2-EF1-copGFP2aPro	6,214	997	1,029	Puromycin
pBsd-GOI-A	Rosa26 (6)	Bsd-gene-of-interest-A	5,637	1,074	4,290	Blasticidin
pNeoR-GOI-B	Rosa26 (6)	NeoR-gene-of-interest-B	4,109	1,095	4,574	Neomycin

Primer sequences used for each genotyping

Figures	Forward	Reverse	Locus
Figure 1F, 5’ KI; Figure 3D, 5’ KI; Figure 4D, Rosa26 5’ KI; Supplemental Figure S2C, 5’ KI	TGGTGGAGCCGTTCTGTGAGACAGC	ACAATAACCAGCACGTTGCCCAGGAG	Rosa26
Figure 1F, 3’ KI; Figure 3D, 3’ KI; Supplemental Figure S2C, 3’ KI	TGCTGCCCGACAACCACTACCTGAG	TAGAGCACAAGCACACACAACACAACAACC	Rosa26
Figure 3F, 5’ KI	GCGCTAACTTCAGTCCTGCCTTCC	CACGACCGGCAAACGGACAGAAG	Stra8
Figure 3F, 3’ KI	AAGCCCGGTGCCTGAAATCAAC	ACTCTCCACTGTGCCTGCCTGTAAC	Stra8
Figure 4D, Rosa26 3' KI	AATATGAGAGAGCTGAGGGCAGAC	TAGAGCACAAGCACACACAACACAACAACC	Rosa26
Figure 4D, Cd6 5' KI	GCAATTTGTCAGAGGAGGAGAGCACC	ACAATAACCAGCACGTTGCCCAGGAG	Cd6
Figure 4D, Cd6 3' KI	GACATCGGCAAGGTGTGGGTCG	GAGGAGTAAGAGGGCGTGGCTACCAG	Cd6
Supplemental Figure 4C, Nanog 5’ KI	ATCTTCTACTTCACCTCCCACACCCATCC	CCATGTTATCCTCCTCGCCCTTGCTC	Nanog
Supplemental Figure 4C, Nanog 3’ KI	GGTGCCACTCCCACTGTCCTTTCC	TGCAGCCTGCCACATCAGGTGATC	Nanog
